# Placebo-Suggestion Modulates Conflict Resolution in the Stroop Task

**DOI:** 10.1371/journal.pone.0075701

**Published:** 2013-10-09

**Authors:** Pedro A. Magalhães De Saldanha da Gama, Hichem Slama, Emilie A. Caspar, Wim Gevers, Axel Cleeremans

**Affiliations:** 1 CO3 - Consciousness, Cognition & Computation Group, Université Libre de Bruxelles (ULB), Brussels, Belgium; 2 UNESCOG - Research Unit in Cognitive Neurosciences, Université Libre de Bruxelles (ULB), Brussels, Belgium; 3 UR2NF - Neuropsychology and Functional Neuroimaging Research Unit, Université Libre de Bruxelles (ULB), Belgium; 4 Department of Clinical and Cognitive Neuropsychology, Erasme Hospital, Université Libre de Bruxelles (ULB), Brussels, Belgium; 5 CRCN - Center for Research in Cognition & Neurosciences, Université Libre de Bruxelles (ULB), Brussels, Belgium; 6 UNI - The ULB Neurosciences Institute, Université Libre de Bruxelles (ULB), Brussels, Belgium; University of California, Davis, United States of America

## Abstract

Here, we ask whether placebo-suggestion (without any form of hypnotic induction) can modulate the resolution of cognitive conflict. Naïve participants performed a Stroop Task while wearing an EEG cap described as a “brain wave” machine. In Experiment 1, participants were made to believe that the EEG cap would either enhance or decrease their color perception and performance on the Stroop task. In Experiment 2, participants were explicitly asked to *imagine* that their color perception and performance would be enhanced or decreased (non-hypnotic imaginative suggestion). We observed effects of placebo-suggestion on Stroop interference on accuracy: interference was decreased with positive suggestion and increased with negative suggestion compared to baseline. Intra-individual variability was also increased under negative suggestion compared to baseline. Compliance with the instruction to imagine a modulation of performance, on the other hand, did not influence accuracy and only had a negative impact on response latencies and on intra-individual variability, especially in the congruent condition of the Stroop Task. Taken together, these results demonstrate that expectations induced by a placebo-suggestion can modulate our ability to resolve cognitive conflict, either facilitating or impairing response accuracy depending on the suggestion’s contents. Our results also demonstrate a dissociation between placebo-suggestion and non-hypnotic imaginative suggestion.

## Introduction

People have always been fascinated by the extent to which belief or will may influence behavior. Many proverbs (e.g., "where there is a will, there is a way") and concepts (e.g., optimistic thinking) reflect this intuition of an important link between one’s dispositions and subsequent behavior. Response Expectancy Theory [1,2,3] posits that we sometimes unintentionally behave so as to produce an outcome that fits our initial expectancies. Such expectancies that a given behavior will result in a particular outcome increase the likelihood that the corresponding behavior occurs, so instantiating a kind of self-fulfilling prophecy [4,5].

In health related domains, hypnosis (i.e., a specific state primarily associated with attentive-receptive absorption and characterized by extreme focused attention and compliance with suggestion [6,7]) and placebo, including medical instruments (e.g., pills, needles, medical coats, stethoscope, medical machineries) and procedures (e.g., surgery, medical exams), can likewise generate or activate expectancies that exert both psychological and physiological effects [8,9,10,11,12,13,14]. Importantly, in both hypnosis and placebo, *suggestion* (i.e. a communication that a participant will experience a particular response [15]) is thought to be the main inductor of behavioral changes, independently of any active substance or component [16]. Thus, suggestion (either intentional or unintentional, direct or indirect, verbal or non-verbal) creates response expectancies that activate automatic responses, which will in turn influence cognition and behavior so as to shape them congruently with the expected outcome [17].

Research has shown that manipulating expectancies can bias different cognitive processes such as the experience of pain [18,19,20], visual awareness [21], memory [22,23], implicit learning [24,25] and emotions [26]. However, despite a large body of research [27], placebo-suggestion influences on conflict resolution and cognitive control, both high-level psychological functions, have been little studied. Here, we demonstrate that a placebo-suggestion can create expectancies that have a significant impact on conflict resolution assessed by objective measures.

Conflict resolution involves cognitive control processes that enable adaptive responses to unusual or conflicting situations. Amongst the procedures used to assess conflict resolution, the Stroop Task [28] remains by far the most studied [29,30]. Typically, participants are asked to name the colors of the ink in which words are displayed. When the words are themselves the names of colors, compatibility effects occur. Thus, when the word RED is displayed in green (an incongruent trial), participants require more time and are less accurate in responding “green” than if the word were neutral (e.g., EDGE) or congruent (e.g., GREEN). Recent studies have investigated whether the Stroop interference effect can be reduced through a *post-hypnotic suggestion* (i.e., a suggestion induced during hypnosis but triggered after the hypnotic episode, during the natural state of wakefulness) that words would appear as “gibberish” [7,31,32,33]). As a result of the post-hypnotic word blindness suggestion, both reduction [31,34,35] or elimination [7,32] of the interference in the incongruent condition were observed (albeit only for highly suggestible participants). Similar results have been obtained in other conflict tasks such as the Simon task [36], the McGurk task [27] and the Eriksen Flanker Task [37]. For instance, in the Flanker Task, participants perform a speeded choice reaction time task to target stimuli (usually letters) that are flanked by distractors. The flanker letters can be the same as the target letter (congruent), different (incongruent) or neutral [38]. A post-hypnotic suggestion to attend to the target letter and to perceive the irrelevant flanker letters as out of focus eliminated the flanker compatibility effect (again in highly suggestible participants only). Post-hypnotic suggestion thus seems to be effective in reducing or eliminating interference effects in highly suggestible participants.

Thus far, manipulations using suggestion to induce the experience of an imaginary state of affairs without prior hypnotic induction (also termed ‘non hypnotic imaginative suggestion’ or simply ‘imaginative suggestion’ [39,40,41,42,43,44]) have produced contradictory results. For instance, in a study by Raz et al. [34], the Stroop effect was successfully reduced in highly suggestible participants who had previously received an imaginative suggestion to perceive Stroop words as “gibberish”. These results indicate that a reduction of the Stroop interference can be accomplished regardless of whether hypnosis is induced. A recent study extended this observation by showing a linear relationship between the reduction of the Stroop interference and hypnotic suggestibility across the whole hypnotizability spectrum [45]. On the other hand, Iani et al. [37] failed to replicate this modulation of conflict resolution. When highly suggestible participants were asked to *imagine* that the target letter was brighter and that the flankers were blurred, less luminous, and further away from focus ( [37], experiment 2), no reduction in conflict resolution was observed. The same pattern of results was observed using the Simon task [36].

However, if one considers Response Expectancy Theory, it is possible that asking participants to imagine a modification of the visual percept (i.e., an instruction to volitionally experience a certain response [15]) fails to induce sufficiently potent expectancies (if any). Indeed, when participants are asked to use their imagination, they are given a direct instruction (or command) to intentionally act as though they experienced a certain behavior. Under such conditions, the actual belief that the percept has changed is thus lacking.

Some non-hypnotic suggestions do not involve an invitation to experience imaginary events but are rather intended to cause the person to believe that the external world has actually changed. For example, a placebo-suggestion can be thought of as a verbal statement that misleads a person into believing that a pharmacologically inert substance has certain chemical properties that it does not truly possess [44]. According to Response Expectancy Theory, hypnotic suggestion, imaginative suggestion and placebo suggestion are all instances of the broader phenomenon of suggestion, and the mechanism responsible for the effects produced by such suggestion is response expectancy [1,2,15]. If true, a non-hypnotic suggestion reinforced by placebos (i.e., a placebo-suggestion) through which participants actually believe the suggestion could create expectations that modify the manner in which participants handle cognitive conflict. Again, if true, such a placebo-suggestion, devoid of any hypnotic connotation (i.e., hypnotic induction or hypnotic suggestibility) should be able to effectively reduce the Stroop interference effect. In contrast, if expectations induced by placebo-suggestion are different from those induced by imaginative suggestion (hypnotic or non-hypnotic), the impact on cognitive conflict resolution might also differ.

A further issue that has not been addressed so far by previous post-hypnotic suggestion studies is whether the influence of suggestion is limited to the reduction of the Stroop interference or whether it can also lead to *increased* interference. Such inverted effects have already been demonstrated in other domains such as pain [46,47,48], implicit learning [24,25] and memory [49]. For instance, the verbal suggestion that very cold water is healthy (vs. unhealthy) modified the perception of experimentally induced pain when participants’ hands are immersed in cold water, according to the positive or negative nature of the suggestion [47]. The fact that reverse effects are obtained in the exact same situation, with an identical induction mechanism of expectancies (a non-hypnotic suggestion) but varying only in the content of the suggestion (positive vs. negative) is a powerful demonstration that the suggestion and subsequent expectancies are responsible for the changes in performance. Additionally, such a pattern of results makes it possible to rule out concurrent hypotheses, for instance an improvement of participants’ performance through mere increased motivation, or a Hawthorn effect (an improvement of the participant’s performance as a result of being under observation), both of which are difficult to disentangle from each other through unidirectional suggestion only.

To address these issues, we conducted two experiments aimed at exploring whether a placebo-suggestion devoid of any hypnotic component is able to effectively modulate conflict resolution in the Stroop Task. The first experiment manipulated participants’ beliefs that a (sham) electroencephalogram (EEG) device is able to enhance (vs. decrease) the ability to perceive colors. A second experiment used an imaginative suggestion and attempted to separate the influence of compliance with the instructions and demand characteristics [50] from the more complex mechanism of placebo, which depends on participants’ beliefs about the suggestion. Participants were asked to imagine (as in the experiment of Iani et al. [37]) a modification of the visual percept. In contrast to placebo-suggestion, the instruction to imagine does not involve believing in the phenomenon.

## Experiment 1

### Methods

**Figure 1:**
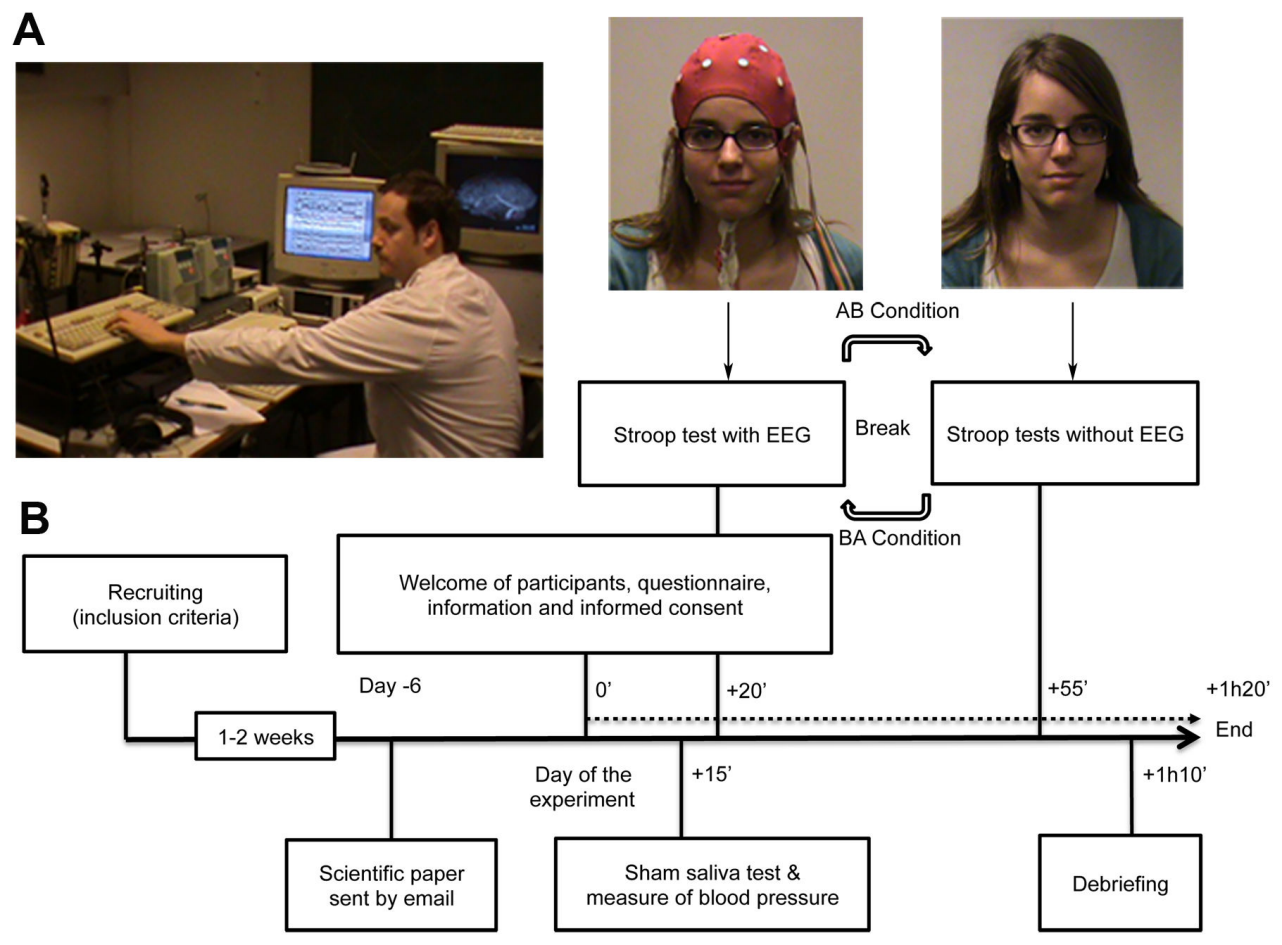
Components of the Placebo-suggestion. (A) Context-placebos (laboratory, equipment, running software and experimenter). (B) Timeline of the different parts of Experiment 1.

The experiment was conducted in accordance with the Declaration of Helsinki and approved by the local ethics committee of the Erasme Hospital, Université Libre de Bruxelles (ULB). Written informed consent was obtained from all participants previous to the study. The individuals in the photograph in Figure 1A have given written informed consent, as outlined in the PLOS consent form, to publication of their photograph appearance.

### Participants

**Figure 2:**
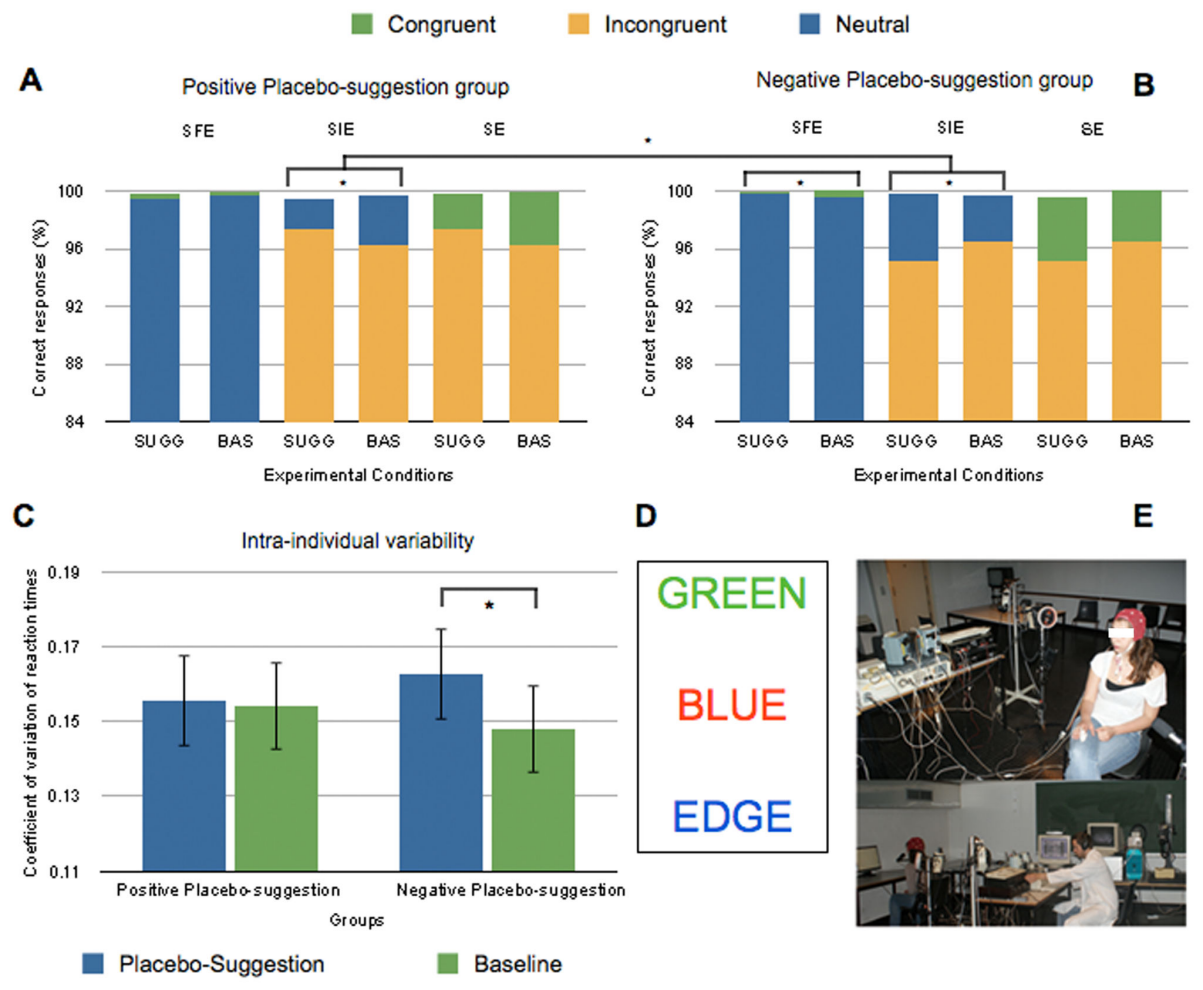
Results of Experiment 1 for accuracy and intra-individual variability.

Two groups of fourteen participants each, matched for age (mean age = 24 y., SD = 6.03, *t*(26) = .874, *p* = .39), gender (4 males in the positive group and 6 males in the negative group, χ^2^(1, N = 28) = .622, *p* = .43), and unscreened for hypnotic suggestibility, were recruited through posters and web announcements, and agreed to participate in exchange for a monetary compensation of €10/h. Participants with past knowledge or direct experience with electroencephalography were excluded from further participation. Participants were told that the purpose of the study was to replicate previous effects about the capacities of a ‘modified electroencephalography machine’ (in reality an inactive electroencephalography system) to modulate participants’ visual ability to perceive colors, (Figure 1A and Figure 2E).

 Accuracy (correct responses - CR) in the three Stroop congruency subtractions (SFE, SIE and SE), under Placebo-suggestion compared to baseline (A) in the positive group and (B) in the negative group. The upper parts of the stacked histogram graphs represent the Stroop congruency subtractions. (C) Intra-individual variability of reaction times in the positive and negative groups under Placebo-suggestion compared to baseline. (D) Examples of Stroop stimuli in the three conditions: congruent, incongruent and neutral, downward. (E) The sham ‘brain wave’ machine and the experimental room.

Note: SFE = Stroop Facilitation effect; SIE = Stroop Interference effect; SE = global Stroop effect. SUGG = suggestion; BAS = Baseline. * indicates a significant difference (p < .05) between conditions. Error bars refer to standard errors.

### Materials

Participants sat at a viewing distance of approximately 60 cm in front of a computer monitor. The stimuli consisted of two classes of French words: six color words (BLEU-blue, BRUN-brown, VERT-green, ROSE-rose, ROUGE-red and JAUNE-yellow), and six neutral words matched in syllable number and lexical frequency (BORD-edge, JUSTE-fair/just, BRUSQUE-sudden/abrupt, VENIR-come, ROUTE-road), displayed in one of the same six colors. Words appeared at the center of the computer screen, where a black fixation cross was visible.

All stimuli were presented against a white background, and the stimuli subtended visual angles of 0.5° vertically, and 1.3° to 1.9° horizontally (depending on word length). Three experimental Stroop conditions were used: a *congruent* condition consisting of a color word inked in its own color (e.g. color word RED displayed in red); an *incongruent* condition consisting of a color word inked in any of the six colors, other than the one to which it referred (e.g. color word RED inked in green) and a *neutral* condition consisting of a neutral word inked in any one of the six colors (e.g. word JUSTE displayed in red) (Figure 2D).

Participants were asked to name the ink color in which the word was written as quickly and as accurately as possible, attempting not to read it. Each trial began with the presentation of a fixation cross (black on a white background) for 250 ms. Then, the fixation cross was immediately replaced by the stimulus (one of the six color words or one of the six neutral words) presented in one of the six possible colors, which remained on the screen until participants responded or for a maximum of 2 seconds. The next trial was initiated 2000 ms after the response. No visual or verbal feedback information concerning participants’ performance was provided. Reaction time latencies were automatically recorded with a vocal key. Response accuracy was recorded manually after each stimulus by the experimenter, following an established list of correct responses. Responses were encoded as correct, erroneous or invalid (e.g., technical failure). Participants performed two blocks of 270 stimuli (90 stimuli by Stroop condition) preceded by a practice block of 10 stimuli.

### Design and Procedure

The experimental design followed a mixed factorial model with Group (positive suggestion vs. negative suggestion) as between-subjects factor and Stroop Condition (congruent, incongruent, neutral) and Suggestion (placebo-suggestion vs. baseline) as within-subject factors. The sham EEG was removed during the positive or negative baseline conditions. Order of the Suggestion factor was counterbalanced across participants.

### Expectancy manipulation (placebo-suggestion)

We manipulated participants’ expectations regarding the properties of a sham EEG machine and its capacity to either enhance (Positive Group) or decrease (Negative Group) participants’ visual ability to perceive colors.

The credibility of the study was potentialized through multiple placebos (context, procedure and objects). Recruiting inclusion criteria (e.g. not consuming stimulant medication, good night of sleep the day before the experiment, no coffee drinking four hours prior to the experiment), a sham saliva test described as detecting non-authorized substances, and a sham measure of participants’ blood pressure (diastolic and systolic, values were always communicated as 12/6) simulated familiar medical routines. The testing room replicated a laboratory and included several machines as well as running software. All devices were disabled but made functional noises and lighting (Figure 1A and Figure 2E).

In the first step of the manipulation, expectations were induced by written information (direct suggestion). A simplified scientific paper that described the properties of the EEG and the (fictitious) scientific background was sent by mail to participants six days before the experiment. The document was seven pages long and was divided into five sections: (1) ‘Information about Electroencephalography’, (2) ‘The original study of the phenomenon explained’, (3) ‘Use of EEG and the present experiment’,(4) ‘The aim of the present experiment’ and (5) ‘Important Reminder’ (Document S1 and Document S2). The first section contained real statements about electroencephalography, while sections two and three induced false scientific background about the sham properties of the EEG and respective effects (positive or negative) found on participants. Finally, sections four and five explained the expected effects of the EEG (positive or negative), according to previous (fictitious) studies. To encourage participants to read the material carefully, participants were told that a comprehension test would be administered before the beginning of the experiment. The information concluded (in section 4 of the scientific paper) as follows for the positive and negative groups, respectively (translated approximately here from French):

“*You will better discriminate* (vs. be *worse at discriminating*) *the displayed stimuli, and your attentional capacity will be improved* (vs. *impaired*). *This will effectively reduce* (vs. *increase*) *the number of errors made during the task*”.

Once participants had arrived at the lab, they first waited in the hallway, where several scientific posters about cognitive psychology studies were displayed (indirect suggestion). Next, participants answered comprehension questions about the document previously sent by email. In order to reinforce previous suggestions, a verbal information (direct suggestion) was briefly repeated immediately before the experiment: the experimenter read a short version of the early document (Document S3 and Document S4). After responding to the comprehension questions and hearing a short exposition of the document, participants provided their written informed consent. The argument used to justify the procedure was that participants had to be fully aware of the material used and the effects expected before they signed the written consent.

The experimenter and his confederate, wearing white medical coats, introduced themselves as the senior scientist and his PhD student. When the sham EEG was placed on participants’ head, a pre-registered electrical activity was displayed on a computer screen and described to participants as their own. This was followed by a simulated adjustment of the electrodes. All participants then completed the Stroop Task with and without the sham EEG. Participants had a 10 minutes break between conditions. In the positive or negative baseline condition, participants were simply asked to name the ink color in which the word was written as quickly and as accurately as possible, attempting not to read it (i.e., classical Stroop Task instructions). After the experiment, a debriefing was held individually. The overall procedure is illustrated in Figure 1B.

## Results

### Data Analysis

Performance in the Stroop Task was measured by participants’ latencies (reaction times - RT), and accuracy (percentage of correct responses). The intra-individual coefficient of variation of RT (Coefficient of variation of RT = RT standard deviation divided by RT mean) was computed as an index of intra-individual variability [51].

Stroop congruency subtractions were also computed. Stroop interference and facilitation effects were assessed as the difference between incongruent and neutral trials (I–N), and between neutral and congruent trials (N–C), respectively; the global Stroop effect is the gross difference between incongruent and congruent trials (I–C) [7] (see Table 1).

**Table 1:** Mean reaction times (RT) and correct responses (CR) for Stroop congruency subtractions as a function of Group and Suggestion in Experiment 1.

		**Baseline**			**Placebo-Suggestion**		
		**SFE (N-C)**	**SIE (I-N)**	**SE (I-C)**	**SFE (N-C)**	**SIE (I-N)**	**SE (I-C)**
PG	RT (ms)	67 (50)	93 (60)	160 (101)	53 (63)	103 (79)	156 (100)
	CR (%)	-0.25 (0.49)	-3.33 (4.60)	-3.58 (.4.79)	-0.32 (0.53)	-2.07 (4.06)*	-2.40 (4.25)
NG	RT (ms)	50 (33)	87 (60)	137 (61)	48 (29)	97 (68)	146 (82)
	CR (%)	-0.41 (0.72)	-3.16 (2.66)	-3.57 (2.95)	0.33 (0.95)*	-4.71 (3.40)*	-4.38 (3.55)

Note. Standard deviations are given in parentheses; PG = positive suggestion group; NG = negative suggestion group; RT (reaction times) are in milliseconds; CR (correct responses) are in percentages; SFE = Stroop Facilitation effect; SIE = Stroop Interference effect; SE = global Stroop effect; * indicates a significant difference (*p* < .05) between suggestion and baseline conditions. For correct responses, a negative value indicates a standard compatibility effect (facilitation or interference).

Data were submitted to a repeated measures ANOVA, with Group (Positive vs. Negative) as a between-subjects factor and with Suggestion (Suggestion vs. Baseline) and Stroop Condition (Congruent, Incongruent and Neutral) as within-subject factors. RT to errors and to trials preceded by an error were discarded. We used a generalized extreme studentized deviate (GESD) test [52] with an r of 20% to drop RT outliers by participant, Suggestion and Stroop Condition (2.2% of the data were discarded). Planned comparisons and Tukey’s post-hocs were performed when appropriate. For direct comparison between suggestion and baseline both in positive and in negative groups, we used one-tailed t-test analyses according to our initial hypotheses. Because accuracy is a variable that is not normally distributed (i.e., it is heavily skewed towards 1), data were analyzed using arcsine transformations to achieve a satisfactory level of normality. Results of transformed data are reported in the main text. For ease of interpretation, the descriptive statistics presented below are untransformed.

### Reaction Times

A repeated measures ANOVA conducted on Reaction times (RT) revealed a main effect of Stroop Condition, *F*(2, 52) = 74.49, *p* < .001. Tukey’s post-hoc tests indicated that congruent trials (705 ms) were faster than both neutral (760 ms, *p* < .001, Stroop facilitation effect = 55 ms), and incongruent trials (855 ms, *p* < .001, global Stroop Effect = 150 ms). Incongruent trials were slower than neutral (*p* < .001, Stroop interference effect = 95 ms). No interaction was observed between Stroop Condition and Suggestion or Group (all *F* values < 1). Stroop congruency subtractions (Table 1) were not influenced by Suggestion, Group, or the interaction between Group and Suggestion (all *F* values < 1).

In sum, RT latencies displayed a classical Stroop pattern, without any influence of the placebo-suggestion (negative or positive).

### Coefficient of Variation of RT

A repeated measures ANOVA conducted on coefficients of variation of RT disclosed a main effect of Stroop Condition (*F*(2, 52) = 29.07, *p* < .001), Suggestion (*F*(1, 26) = 5.94, *p* = .022), and a significant interaction between Suggestion and Group (*F*(1, 26) = 4.36, *p* = .047).

For Stroop Condition, Tukey’s post-hoc tests indicated that incongruent trials (.18) were more variable than neutral (.138, *p* < .001) and congruent trials (.148, *p* < .001), which were identical (*p* = .21).

Post-hoc tests were also computed for the interaction between Suggestion and Group. In the negative group, participants’ intra-individual variability increased under suggestion (.163) compared to baseline (.148, *p* = .018), while no difference was observed in the positive group between suggestion and baseline conditions (*p* = .995) (Figure 2C).

To summarize, participant’s intra-individual variability displayed a classical Stroop pattern. Variability was influenced by placebo-suggestion with participants being more variable when told that the EEG would decrease their color perception. On the opposite, variability was not modulated when participants were told that the EEG would increase their color perception.

### Accuracy (percentage of correct responses)

Concerning percentages of correct responses, a repeated measures ANOVA conducted on transformed data (arcsine) disclosed a main effect of Stroop Condition (*F*(2, 52) = 78.72, *p* < .001), a significant interaction between Suggestion and Group (*F*(1, 26) = 5.8, *p* = .023), and between Stroop Condition, Suggestion and Group, *F*(2, 52) = 5.42, *p* = .007 (Figure 2A and Figure 2B).

Tukey’s post-hoc tests for Stroop Condition indicated that accuracy was lower for incongruent (96.3%) than for neutral (99.6%, *p* < .001, Stroop interference effect = -3.3%) and congruent (99.8, *p* < .001, global Stroop effect = -3.5%) trials, which were identical (*p* = .954).

Stroop congruency subtractions were computed to decompose the interaction between Stroop Condition, Suggestion and Group.

Analyses of the Stroop interference effect indicated a marginal main effect of Group, *F*(1, 26) = 4.093, *p* = .053, and a significant interaction between Group and Suggestion (*F* = 7.91, *p* = .009). This interaction revealed a decreased interference (-1.26%) in the positive group under suggestion compared to baseline (*t*(14) = -1.98, *p* = .035). Inversely, interference increased (1.55%) in the negative group under suggestion compared to baseline (*t*(14) = 2, *p* = .033).

Stroop facilitation effect was influenced by the interaction between Group and Suggestion, *F*(1, 26) = 4.36, *p* = .047. Planned comparisons indicated that, Stroop facilitation effect was lower under negative suggestion (0.3%) compared to baseline (-0.4%) in the negative group (*F*(1, 26) = 6.92, *p* = .007), whereas no difference was observed between conditions in the positive group (*F* < 1).

The global Stroop effect was not influenced by Suggestion, or Group, or by the interaction between Group and Suggestion (Table 1).

To summarize, participants exhibited less interference from written color words when the EEG was thought to be active and described as enhancing participants’ ability to perceive colors. On the opposite, participants showed more interference when the EEG was thought to be active and described as decreasing color perception. Further, the facilitation from congruent color words was decreased under negative placebo-suggestion but was not modified by positive placebo-suggestion.

### Discussion of Experiment 1

A modulation of conflict resolution in the Stroop Task was observed on accuracy but not on participants’ RT latencies. The positive group showed decreased interference compared to baseline, while the negative group showed increased interference. Stroop facilitation effect was also modulated by placebo-suggestion, showing decreased facilitation in the negative group only. In addition, participants’ intra-individual variability of RT increased under placebo-suggestion, again in the negative group only.

Thus, our results indicate that cognitive conflict resolution can be modified as a function of expectancies related to participants’ belief towards a given phenomenon.

Nevertheless, it can also be argued that results of Experiment 1 can be attributed to participants’ commitment to please the experimenter, similar to *demand characteristics* [50]. Indeed, given the fact that our placebo-suggestion provided the expected behavior (“*This will effectively reduce* (vs. *increase*) *the number of errors made during the task*”), the present results could have been influenced by compliance with the instructions. The concept of *demand characteristics* suggests that participants in experimental settings continuously and consciously attempt to reconstruct the experimenter’s hypotheses based on available cues and on their understanding of how participants are expected to behave [50]. Demand characteristics states that cues which govern participants’ perception include both implicit and explicit information such as instructions, subtle cues provided by the experimenter, the experimental procedure and the experimenter himself [53]. Noticeably, although the use of explicit instructions may represent clear information regarding the participant expected behavior and the study hypothesis (as in our experiment), the “demand characteristics” hypothesis considers that more covert or subtler cues (as used in usual hypnotic-suggestion situations) may nevertheless be more powerful than overt instructions [53].

In an attempt to separate the impact of expectancies created by placebo-suggestion from the influence of *demand characteristics* [50] and of compliance with the instructions, we applied the procedure used by Iani et al. [37], asking participants to *imagine* the expected behavior. In contrast to placebo-suggestion, asking participants to imagine an expected behavior does not involve believing in the phenomenon. Here, the direct imaginative suggestion about the expected behavior is thus similar to ‘role-playing participants’ [53] trying to simulate the expected behavior described in the placebo-suggestion of Experiment 1 [54].

## Experiment 2

Experiment 2 investigated whether the results of Experiment 1 were a mere product of compliance with the instructions or resulted instead from response expectancy (i.e. placebo-suggestion). If the former is true, then giving the same instructions concerning the expected behavior to participants (i.e. asking participants to deliberately and consciously imagine that their capacity to perceive colors is enhanced or impaired) without belief induction should yield the same results as observed in Experiment 1. For the sake of conciseness, we call the deliberate attempt to imagine a phenomenon *imaginative suggestion*.

### Methods

As in Experiment 1, written informed consent was obtained from all participants previous to the study.

### Participants

Two groups of fourteen participants each, matched for age (mean age = 19 y., SD = 1.97, t(26) = -.472, *p*=.641), gender (4 males in the positive group and 3 males in the negative group, χ^2^(1, N = 28) = .19, *p* = .663) and unscreened for hypnotic suggestibility were recruited through posters and agreed to participate in exchange for course credits. Participants were told that the purpose of the study was to study the impact of imagination to modulate participants’ visual ability to perceive colors. Inclusion criteria were similar to those in Experiment 1. The original sample comprised 31 participants but three participants were discarded after not respecting inclusion criteria.

### Materials

The same materials as in Experiment 1 were used. Specifically, participants were tested in the same room (the sham laboratory), by the same experimenter, and with the same material as in Experiment 1, but without the sham EEG.

### Design and Procedure

The experimental design followed the same mixed factorial model as in Experiment 1 with Group (positive vs. negative) as between-subject factor, Stroop Condition (congruent, incongruent, neutral) and Imaginative suggestion (imaginative suggestion vs. baseline) as within-subject factors. No instruction regarding the use of imaginative suggestion was made during the positive or negative baseline conditions. Order of the Imaginative suggestion factor was counterbalanced across participants.

### Imaginative suggestion instructions

In Experiment 2, we applied the procedure of Iani et al. [37]. Participants were explicitly asked to try to *imagine* their ability to perceive colors as being enhanced (positive group) or impaired (negative group). The main sentences and instructions (direct suggestion) were the same as in Experiment 1. However, written or verbal information concerning a placebo capable of modulating color perception was not given to participants. The following instruction was verbally presented to both positive and negative groups, respectively (translated approximately here from French):

“*In this experiment you will perform a computerized task in which you will be asked to discriminate colors. Your speed and performance will be measured.*

*To help you focus your attention, we ask you to try to imagine that your ability to perceive colors is* *improved* (vs. *deteriorated*) *and that the color differences are* *highly* *perceptible* (vs. *barely perceptible*)*. Imagine that you have the capacity* *to* *improve* (vs. *deteriorate*) *your speed and response accuracy in tasks involving color visibility. Imagine that you will* *better* *discriminate* (vs. *be worse at discriminating*) *the displayed stimuli, and that your* *attentional* *capacity* *will* *be* *improved* (vs. *impaired*)*. Imagine that you will be able to respond* *quickly* (vs. *slowly*) *and with* *great* *precision* (vs. *less precisely*)*.* *Imagine* *that* *this* *will* *effectively* *reduce* (vs. *increase*) *the* *number* *of* *errors* *made* *during* *the* *task*. *Now try to imagine it and we will begin the task*”

In the positive or negative baseline condition, participants were simply asked to name the ink color in which the word was written as quickly and as accurately as possible, attempting not to read the color words (i.e., usual Stroop Task instructions). Participants had a 10 minutes break between conditions. After the experiment, a debriefing was held individually.

## Results

### Data Analysis

The same analyses were performed as for Experiment 1. Concerning outliers, 2.92% of the data were discarded.

### Reaction Times

A repeated measures ANOVA conducted on reaction times (RT) in the Stroop Task disclosed a main effect of Stroop Condition (*F*(2, 52) = 126.17, *p* < .001), Group (*F*(1, 26) = 16.53, *p* < .001) and Imaginative suggestion (*F*(1, 26) = 11.14, *p* = .002).

**Figure 3:**
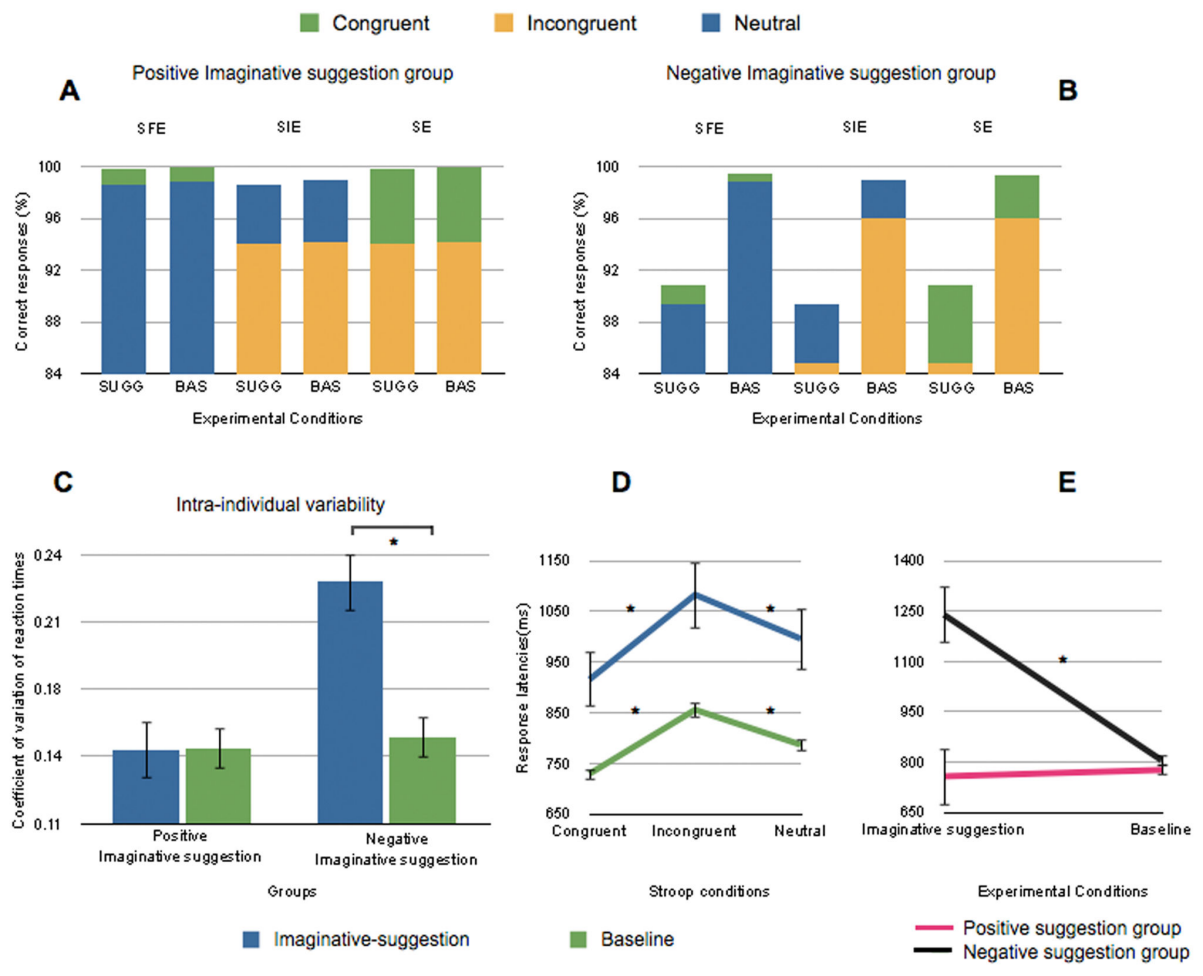
Results of Experiment 2 for accuracy, intra-individual variability and response latencies. Accuracy (correct responses - CR) in the three Stroop congruency subtractions (SFE, SIE and SE), under Imaginative suggestion compared to baseline (A) in the positive group and (B) in the negative group. The upper parts of the stacked histogram graphs represent the Stroop congruency subtractions. (C) Intra-individual variability of reaction times in the positive and negative groups under Imaginative suggestion compared to baseline. (D) Response latencies (Reaction times – RT) in the three Stroop conditions (congruent, incongruent and neutral) under Imaginative suggestion compared to baseline, and (E) in the positive group and in the negative group under Imaginative suggestion compared to baseline. Note: SFE = Stroop Facilitation effect; SIE = Stroop Interference effect; SE = global Stroop effect. SUGG = suggestion; BAS = Baseline. * indicates a significant difference (p < .05) between conditions. Error bars refer to standard errors.

The interaction between Imaginative suggestion and Group (*F*(1, 26) = 13.26, *p* < .001) was significant. Tukey’s post-hoc tests revealed no difference between Imaginative suggestion (757 ms) and baseline (776 ms, *p > .1*) in the positive group, but participants in the negative group were slower under imaginative suggestion (1239 ms) compared to baseline (805 ms, *p* < .001) (Figure 3E).

The interaction between Stroop Condition and Imaginative suggestion (*F*(2, 52) = 4.26, *p* = .019) was also significant (Figure 3D). Congruency subtractions (Table 2) revealed that both the Stroop facilitation effect and the global Stroop effect were more pronounced under imaginative suggestion compared to baseline (Stroop facilitation effect = 81 ms vs. 57 ms, *F*(1, 26) = 4.65, *p* = .04; global Stroop effect = 168 ms vs. 126 ms, *F*(1, 26) = 5.5, *p* = .027). There was no significant difference in the Stroop interference effect between Imaginative suggestion and baseline (*F*(1, 26) = 1.84, *p* > .1).

**Table 2:** Mean reaction times (RT) and correct responses (CR) for Stroop congruency subtractions as a function of Group and Imaginative suggestion in Experiment 2.

		Baseline			Imaginative suggestion		
		**SFE (N-C)**	**SIE (I-N)**	**SE (I-C)**	**SFE (N-C)**	**SIE (I-N)**	**SE (I-C)**
PG	RT (ms)	50 (32)	57 (47)	108 (65)	64 (36)	68 (39)	132 (60)
	CR (%)	-1.03 (1.11)	-4.76 (3.98)	-5.79 (4.18)	-1.27 (1.22)	-4.52 (3.53)	-5.79 (3.09)
NG	RT (ms)	64 (36)	81 (45)	145 (56)	97 (76)	106 (73)	203 (118)
	CR (%)	-0.48 (1.43)	-2.86 (3.59)	-3.33 (3.77)	-1.51 (4.86)	-4.60 (13.08)	-6.11 (15.96)

Note. Standard deviations are given in parentheses; PG = positive suggestion group; NG = negative suggestion group; RT (reaction times) are in milliseconds; CR (correct responses) are in percentages; SFE = Stroop Facilitation effect; SIE = Stroop Interference effect; SE = global Stroop effect. No difference reached significance. For correct responses, a negative value indicates a standard compatibility effect (facilitation or interference).

Finally, the interaction between Stroop Condition and Group (*F*(2, 52) = 4.36, *p* =.018) also reached significance. Stroop congruency subtractions (Table 2) revealed that the global Stroop effect was stronger in the negative group (174 ms) compared to the positive group (120 ms, *F*(1, 26) = 5.11, *p* = .032), as was the Stroop interference effect (negative group = 94 ms, positive group = 63 ms, *F*(1, 26) = 4.25, *p* = .049). There was no difference for the Stroop facilitation effect between positive and negative groups (*F*(1, 26) = 2.52, *p* > .1).

Importantly, no significant interaction was observed between Stroop Condition, Imaginative suggestion and Group (*F* < 1).

To summarize, participants’ response latencies were slowed by the request to imagine a decreased color perception but no speedup was produced by the opposite demand. This slowing was not influenced by Stroop interference or facilitation. Imaginative suggestion (positive or negative) also had a general facilitation effect but no impact on interference.

### Coefficient of Variation of RT

A repeated measures ANOVA conducted on the coefficients of variation of Reaction Times (RT) revealed a main effect of Imaginative suggestion (*F*(1, 26) = 13.4, *p* = .001), Group (*F*(1, 26) = 9.28, *p* =.005) and Stroop Condition (*F*(2, 52) = 15.27, *p* < .001). Tukey’s post-hoc tests indicated a classical Stroop pattern with congruent trials (.168) that were more variable than neutral (.154, *p* = .029), and incongruent trials (.182, *p* = .01). Incongruent trials were more variable than neutral trials (*p* < .001).

A significant interaction was observed between Imaginative suggestion and Group (*F*(1,26) = 14.00, *p* <.001), between Stroop Condition and Imaginative suggestion (*F*(2, 52) = 4.84, *p* =.012), and between Stroop Condition, Imaginative suggestion and Group (*F*(2,52) = 5.25, *p* = .008). Tukey’s post-hocs revealed no difference in intra-individual variability between Imaginative suggestion and baseline for all Stroop conditions in the positive group. Contrarily, in the negative group, intra-individual variability was higher under imaginative suggestion compared to baseline for congruent trials (.244 vs. .144, *p* < .001), incongruent trials (.222 vs. .178, *p* = .001) and neutral trials (.215 vs. .134, *p* < .001). Planned comparisons indicated that the increased interference in the negative group under imaginative suggestion, compared to the positive group, was higher for congruent trials than for incongruent trials (*F*(1, 26 = 7.93, *p* = .009). No difference was present between neutral and congruent, or incongruent trials (all *p* values > .05) (Figure 3C).

In sum, intra-individual variability was higher in all Stroop conditions when participants were requested to imagine a diminished color perception. Furthermore, the increase in variability was greater for congruent trials than for incongruent trials. The request to imagine an enhancement in color perception had no impact on intra-individual variability.

### Accuracy (proportion of correct responses)

A repeated measures ANOVA conducted on accuracy revealed a main effect of Stroop Condition (*F*(2, 52) = 52.57, *p* < .0001). Tukey’s post-hoc tests revealed that participants made less correct responses for incongruent (92.2%) than for neutral (96.4%, *p* < .001; Stroop interference effect = -4.2%) and congruent (97.5%, *p* < .001; global Stroop effect = -5.3%) trials, which were not significantly different (*p* = .53; Stroop facilitation effect = -1.1%).

A significant interaction between Stroop Condition and Group (*F*(2,52) = 3.74, *p* = .03) was present (Figure 3A and Figure 3B). Stroop congruency subtractions revealed no difference in accuracy for the Stroop interference effect between positive and negative groups (*F*(1, 26) = 1.59, *p* = .219). The difference in the Stroop facilitation effect between positive and negative groups was marginally significant (*F*(1, 26) = 4.05, *p* = .055). The global Stroop effect was higher for the positive group (-5.8%) compared to the negative group (-4.7%, *F*(1, 26) = 5.9, *p* = .022) (Table 2).

Contrary to Experiment 1, no interaction was found between Group, Stroop Condition and Imaginative suggestion (*F* < 1). This null result suggests the absence of an oriented impact of imaginative suggestion (positive or negative according to the direction of the instruction) on Stroop interference. Thus, the effect of a placebo-suggestion, observed in Experiment 1, seems to differ from compliance with the instructions and imaginative suggestion. However, null results are difficult to interpret through orthodox statistics (e.g., ANOVA) and cannot in and of themselves be used to assert the null hypothesis that requesting participants to imagine a modulation of color perception has no impact on Stroop interference.

To overcome this limitation, we further analyzed the results in both positive and negative groups using the Bayes Factor (Dienes, 2008b, 2011) [55,56]. A Bayes Factor compares two theories. In the positive group, it compares the null hypothesis (H0) that imaginative suggestion is equivalent to the baseline condition in term of modulating the Stroop interference effect, and the alternative hypothesis (H1) that imaginative suggestion suppresses the Stroop interference effect. The Bayes Factor is a number between 0 and infinity, where values greater than three indicate strong evidence for the alternative hypothesis (H1), values less than 1/3 indicate strong evidence for the null (H0), and values between 1/3 and 3 indicate lack of sensitivity [56]. Assessing the sensitivity of the null result depends on specifying what range of effect sizes could be expected if there were effects of imaginative suggestion on the Stroop interference effect. In this case, a maximal effect of Imaginative suggestion should eliminate the Stroop interference effect (Stroop interference effect of Imaginative suggestion = 0). Thus, the difference between the baseline condition and the Imaginative suggestion condition should be equal to the baseline condition (baseline - Imaginative suggestion = baseline -0 = baseline). In contrast, H0 postulates that the effect of Imaginative suggestion is null and that the Stroop interference effect under Imaginative suggestion is equal to the Stroop interference effect in the baseline condition (baseline - Imaginative suggestion = baseline -baseline = 0). With these assumptions in place and using the Dienes’ web calculator [55] on arcsine transformations (as in the orthodox statistics), we thus computed the sample standard error (0.068) and the sample mean (-0.016) of the difference between the baseline and the Imaginative suggestion conditions (values are rounded to three decimals in the text). The upper bound is “0” (indicating no difference between Imaginative suggestion and baseline conditions) and the lower bound is the mean Stroop interference effect in the baseline condition (-0.299). The Bayes Factor was 0.35 in this case. On this basis (i.e., an empirical Bayes factor of nearly 1/3), we can thus conclude that there is positive evidence for the null hypothesis. Thus, we can exclude compliance with the instructions (or imaginative suggestion) as inducing a positive effect on Stroop interference.

In the negative group, the Bayes Factor compares the null hypothesis (H0) that imaginative suggestion is equivalent to the baseline condition in term of modulating the Stroop interference effect, and the alternative hypothesis (H1) that Stroop interference effect is increased under imaginative suggestion compared to baseline. We computed the sample standard error (0.065) and the sample mean (-0.013) of the difference between the baseline and the Imaginative suggestion conditions. The lower bound is thus “0”, meaning that the Stroop interference effect under imaginative suggestion is similar than in the baseline condition. For the upper bound, to keep the same difference between lower and upper bound than for the positive group, we fixed a value equal to the negative value of the mean Stroop interference effect in the baseline condition (0.195), indicating that the Stroop interference effect is two times larger than in the baseline condition (baseline - suggestion = baseline -2*baseline = - baseline). With these assumptions, we observed a Bayes Factor of 0.35. Again, this is positive evidence for the null hypothesis. Thus, we can exclude imaginative suggestion as inducing a negative effect on Stroop interference.

Finally, to compare Experiments 1 and 2 through the Bayesian approach, we also conducted the same analysis on the results of Experiment 1 (in both positive and negative groups). Here, the idea was to assess the alternative hypothesis (H1) that the Stroop interference effect is reduced in the positive group and increased in the negative group under placebo-suggestion compared to baseline, coherently with the results obtained through orthodox statistics.

In the positive group, we computed the sample standard error (0.067) and the sample mean (-0.134) of the difference between the baseline and the placebo-suggestion conditions. The upper bound is “0” (indicating no difference between baseline and placebo-suggestion conditions) and the lower bound is the mean Stroop interference effect under the baseline condition (-0.276). The Bayes Factor was 4.21 (higher than 3), indicating strong evidence for the impact of positive placebo-suggestion on Stroop interference, congruently with the results obtained through orthodox statistics, which were also significant (*p* = .035).

In the negative group, we computed the sample standard error (0.057) and the sample mean (0.114) of the difference between baseline and placebo-suggestion conditions. Here, the upper bound is 0.264 (indicating that the Stroop interference effect is two times larger in the placebo-suggestion compared to the baseline condition) and the lower bound is “0” indicating no difference between conditions. The Bayes factor was 3.94 (higher than 3), indicating, again, evidence that the negative placebo-suggestion increases Stroop interference, congruently with orthodox statistics (*p* = .033).

### Conclusions of Experiment 2

In Experiment 2, compliance with the instructions was investigated as a potential confounding factor for the results of Experiment 1. We aimed to determine if the modulation of conflict resolution in the Stroop Task observed in Experiment 1 resulted from placebo-suggestion or was instead just a product of demand characteristics or compliance with the instructions. Participants in Experiment 2 were instructed to *imagine* their capacity to perceive colors as being enhanced (positive group) or impaired (negative group). Instructions had a strong influence on reaction times when participants were asked to imagine that their performance would be worse. Participants easily followed this instruction. Indeed, results disclosed a strong detrimental impact of instructions on response latencies and intra-individual variability. However, accuracy was not modulated by the instructions. Importantly, participants were not able to improve their performance on the basis of imaginative suggestion. This clearly dissociates from the placebo-suggestion in Experiment 1 where both a decrease and an increase in performance was observed.

In contrast to Experiment 1, intra-individual RT variability was particularly increased by instructions in the negative group for congruent trials. This result probably indicates that participants in the negative group were trying to outperform according to the instructions, which is difficult in congruent trials because of the facilitation effect.

## Discussion

Previous research has highlighted the importance of both hypnosis and suggestibility to induce expectations that modulates conflict resolution, especially in the Stroop and Flanker tasks [34,37]. The present study investigated whether non-hypnotic, expectation-based mechanisms such as placebo-suggestion are sufficient to elicit similar effects in the general population (i.e., a random sample of participants who weren’t screened for hypnotic suggestibility). We also investigated whether this modulation is limited to the suppression of the Stroop interference effect, as previously observed, or whether the Stroop interference effect could also be increased.

Results indicate that, congruently with previous studies using a post-hypnotic suggestion or imaginative suggestion only [34], cognitive conflict can be modified as a function of expectancies. The fact that our non-hypnotic suggestion (reinforced by a context-placebo) modulates conflict resolution diverges from other results [37] and suggests that a simple verbal instruction to *imagine* a modification of cognitive processes is insufficient to induce expectancies capable of modulating cognitive conflict. Participants’ belief in the phenomenon thus appears to be necessary to activate expectancies that the percept has changed and to cause a corresponding effect on effective behavior. This observation has implications for further studies manipulating expectations in a non-hypnotic procedure and highlights the need for both the credibility and the plausibility of the suggestion, which have both been reported as being specific-context dependent (e.g., treatment characteristics, health-care setting, patient’s characteristics, or practitioner’s characteristics) [57,58], and influenced by personal past experience and knowledge [12,14,59]. In Experiment 2, the absence of benefits of a direct imaginative suggestion on Stroop interference in the positive group replicates previous results [37] and is coherent with the particular importance of believing in the phenomenon and expecting his occurrence. Furthermore, the different pattern of performance in the negative group, especially in RT, with an undifferentiated negative impact of instructions on all Stroop conditions, indicates that results of Experiment 1 are not simply attributable to compliance with instructions or demand characteristics. For instance, in contrast to Experiment 1, participants in the negative group were more disturbed by congruent than by incongruent trials, with an increased intra-individual variability indicating that they were trying to outperform according to the instructions, which is difficult in congruent trials because of the facilitation effect. Taken together, our results suggest a dissociation between placebo-suggestion and direct imaginative suggestion.

Contrary to post-hypnotic suggestion studies, our induction of expectancies by a placebo-suggestion does not involve hypnosis or participants exhibiting a high degree of suggestibility, showing that neither feature is necessary to induce cognitive conflict modulation. However, the finding that placebo-suggestion influences accuracy and RT intra-individual variability, but not RT latencies per se, contrasts with findings using post-hypnotic suggestion and non-hypnotic imaginative suggestion and may indicate that imaginative suggestion (with or without hypnotic induction) and placebo-suggestion modulate distinct aspects of conflict resolution.

Recent research studying beliefs about cognitive fatigue (i.e., the cognitive limited resources that are available [60]) suggests that, in some cases, cognitive fatigue (also termed ‘ego depletion’) may result not from a true lack of cognitive resources after an exhausting task, but from people’s beliefs about the effects of mental exertion. The authors assessed participants’ implicit beliefs about two opposite theories indicating either limited-cognitive resources (limited-resource theory) or nonlimited cognitive resources (nonlimited-resource theory). Participants then performed a Stroop Task after completing two conditions: with (ego depletion) or without exhaustion (nondepleting condition). Importantly, the authors used the probability of mistakes on incongruent trials in the Stroop Task as a marker of cognitive fatigue. Only participants with a limited-resource theory showed increased probability of mistakes after exhausting tasks and fatigue induction. Participants with a nonlimited-resource theory showed no difference between the depleting and nondepleting conditions. These results suggest that beliefs are able to modulate the impact of fatigue on conflict resolution, and that the effect is observed on errors. Thus, again, beliefs appear to play an important part in expectancies induction and consecutive modulation of performance, at the error level, in cognitive control tasks.

In addition, recent findings have suggested that hypnosis modulates the Stroop effect at the response level and not at the stimulus level [33], though Raz and colleagues found diminished activation in perceptual areas when the post-hypnotic suggestion was given, indicating a strong modulation of early occipital cortex activity [31]. Because our suggestion focused on target processing (the ink’s color) and not on distractor processing (reading), it might reflect a more stimulus-driven conflict resolution. Furthermore, given that the response modality was verbal in our experiment, associations between stimuli and responses (S-R mapping) were stronger than in post-hypnotic suggestion studies using manual responses, possibly causing additional difficulty to elicit conflict modulation at the response latencies level. Another explanation for this observation is that we mainly emphasized response accuracy (“*This will effectively reduce* (vs. *increase*) *the number of errors made during the task*”), instead of speed of responding. Future approaches could emphasize speed to investigate whether this would specifically modulate speed of responding. Overall, expectations about target processing appear to be as potent in influencing conflict as the distractor-driven (reading) modulators previously revealed, but this modification might also have contributed to the observed differences with post-hypnotic suggestion studies. We hypothesized that, in placebo-suggestion, to draw participants’ attention to the distractor (reading) might lead to ironic effects and increased interference [61]. Apparently, this ironic effect is not present with post-hypnotic suggestion or non-hypnotic imaginative suggestion but still need to be investigated in placebo-suggestion. Importantly, future studies comparing imaginative suggestion (hypnotic or non-hypnotic) and placebo-suggestion should match their procedures to allow direct comparisons between the different methods of expectancies induction.

To summarize, although the psychological link between response expectancy and an expected response continues to be debated, variables such as attitudes, faith, beliefs, hope, and anxiety reduction have all been proposed [10,15,62,63]. Suggestion represents the initial trigger capable of inducing expectancies, which in turn will produce an outcome that fits those initial expectancies. This sequence of events, which casts response expectancy as the final link in the causal chain between suggestion and response (in both hypnosis and placebo phenomena), is an alternative mechanism that applies to both hypnotic and placebo responses. However, whether expectation is the critical determinant [1,64,65] or whether it produces a more nuanced influence on hypnotic effects [27,66,67] is a matter of continuing discussion [43,68]. Therefore, hypnotic suggestion and placebo-suggestion might differ in their mechanisms (e.g., hypofrontality in hypnosis [69,70]) and consecutive influences on behavior. For instance, in the cold control theory of hypnosis [67], imaginative suggestion, with or without hypnotic induction, does not work through expectation as the final mediator, but rather through the intention to perform a motor or cognitive action without awareness of this intention. In this case, imaginative suggestion would be different from placebo-suggestion, as we observed, if placebo-suggestion is mediated by expectations.

Our second hypothesis was that expectations have the potential to decrease Stroop interference but should also lead to enhanced interference according to the direction of the suggestion. Present results have confirmed the capacity of placebo-suggestion to modulate Stroop effect in both directions with reduced interference under positive suggestion and increased interference under negative suggestion, in accordance with our predictions. Facilitation was also significantly decreased under suggestion in the negative group but data inspection suggest possible ceiling effects on accuracy in the congruent and neutral conditions that may have reduce the facilitation effect. Nevertheless, supporting the idea of a more general cognitive disturbance in the negative group, participant’s intra-individual variability showed a significant increase under negative suggestion compared to baseline, while participants in the positive group stayed cognitively stable (Figure 2C). Past research has shown that coefficient of variation of RT is associated with a decrease in consistency in tasks requiring the recruitment of executive control processes such as the Stroop Task [71], and with an impaired capacity for goal maintenance and action monitoring, probably revealing an increased neural noise due to structural, functional, or neuromodulatory brain changes [72,73,74]. Recent studies [75] have indicated that intra-individual variability in RT is a marker of a modulation of the default-mode network, and an intrusion of introspective attentional orientation (task-negative elements) during task-oriented situations (task-positive elements). Thus, the negative suggestion appears to elicit a general impairment in performance monitoring and consistency, modulating the balance between introspective and task-oriented attention, possibly linked to anxiety or stress due to the nocebo-like suggestion [76]. Thus, in contrast to the placebo effect observed in the positive group, the nocebo effect does not specifically impact cognitive conflict, but appears to influence a more diffuse mechanism that possibly involves other cognitive processes (e.g., action monitoring or goal maintenance). Noticeably, our results illustrate the importance of measuring participant’s intra-individual variability when studying the impact of expectancies on cognitive control (or in the study of consciousness) because it represents an oft-neglected sensitive marker [77].

## Conclusion

To conclude, the present study indicates that opposite expectancies, induced through a placebo-suggestion devoid of any hypnotic connotation, are able to both reduce and enhance interference effects in the Stroop Task. Placebo-suggestion also causes a stronger modulation of cognitive conflict than imaginative suggestion. Our results can thus claim a significant contribution to the study of the influence of suggestion and expectations on cognitive processes, specifically cognitive conflict, extending such evidences in the domain of placebo research. Additionally, the present study highlights the possibility that the effects of post-hypnotic suggestion and imaginative suggestion previously observed in the Stroop task [7,34], can also be modulated, to some extent, by beliefs and expectations, regardless of hypnotic suggestibility.

In a broader perspective, our results imply that self-help procedures such as positive thinking or optimistic autosuggestion; personality traits such as self-esteem; or phenomena such as the Pygmalion effect (i.e., the fact that, in hierarchical social relationships expectations for subordinate performance can unconsciously affect leader behavior and subordinate performance [78]), likewise all have the potential to exert detectable effects in conflict situations that require cognitive control.

## Supporting Information

Document S1 **Positive Placebo-suggestion information used in Experiment 1.** Written information (i.e., direct suggestion) describing the properties of the EEG and the (fictitious) scientific background. The document was seven pages long and was divided into five sections. The document was sent by mail to participants six days before the experiment.
(DOCX)

Document S2 **Negative Placebo-suggestion information used in Experiment 1.** Written information (i.e., direct suggestion) describing the properties of the EEG and the (fictitious) scientific background. The document was seven pages long and was divided into five sections. The document was sent by mail to participants six days before the experiment.
(DOCX)

Document S3 **Positive Placebo-suggestion information read to participants in Experiment 1.** Verbal information (i.e., direct suggestion) was a short version of the early document previously sent by mail (Document S1). In order to reinforce previous suggestions, verbal information was briefly repeated immediately before the experiment.
(DOCX)

Document S4 **Negative Placebo-suggestion information read to participants in Experiment 1.** Verbal information (i.e., direct suggestion) was a short version of the early document previously sent by mail (Document S2). In order to reinforce previous suggestions, verbal information was briefly repeated immediately before the experiment.
(DOCX)
