# Supplementary material for: Placebo-Suggestion Modulates Conflict Resolution in the Stroop Task
Source: PLoS One. 2013 Oct 9;8(10):e75701. doi: 10.1371/journal.pone.0075701 (PMC3794044; doi:10.1371/journal.pone.0075701)
Supplement: Document S2 — Negative Placebo-suggestion information used in Experiment 1. Written information (i.e., direct suggestion) describing the properties of the EEG and the (fictitious) scientific background. The document was seven pages long and was divided into five sections. The document was sent by mail to participants six days before the experiment. (DOCX) [file pone.0075701.s002.docx]

**Supplementary material**

**(DOCUMENT S2)**

Pedro A. Magalhães De Saldanha da Gama, Hichem Slama, Emilie A. Caspar,

Wim Gevers & Axel Cleeremans

The following text is the text used in experiment 1. Translated (approximately) here from the original French version. The original French version can be made available upon demand to the following address:

Pedro Magalhães De Saldanha da Gama

Université Libre de Bruxelles Cp 191BR, Av. F.-D. Roosevelt, 50, 1050 Bruxelles, Belgium

Telephone number: +32 2 650 49 19

E-mail address: [pmagalha@ulb.ac.be](mailto:pmagalha@ulb.ac.be)

**DOCUMENT S2**

**EXPERIMENT 1**

(Negative Placebo-suggestion group: *written* information document)

*Please read this document carefully!*

**Information about Electroencephalography**

The invention of electroencephalography is generally attributed to Hans Berger, a German physiologist who began studying it in the 1920s and recorded the first signal of brain activity in 1929. His work was continued by the British scientist Edgar Douglas Adrian, who received the Nobel Prize for Physiology in 1932.

Figure 1*. Photo of an electroencephalography cap.*

Electroencephalography (EEG) is a method of cerebral examination that measures the brain’s electrical activity via electrodes that are attached to the scalp. These electrodes transmit a signal that can be represented as a line graph, called an electroencephalogram. EEG is painless and non-invasive, and provides information on the neurophysiological activity in the brain over time, particularly the cerebral cortex, either to aid neurological diagnoses, or in cognitive neuroscientific research. The signal measured by EEG is the sum of all the synchronous post-synaptic action potentials of a large number of neurons.

Given that the electrical signal generated by neurons is very weak, the electrical potential measured at the scalp needs to be amplified.

Figure 2. *An electroencephalogram: electrical activity measured by electroencephalography (EEG)*

Recent studies (by Magaldanha et al., 2011 *in press*) have shown that EEG can temporarily influence color discrimination and visual recognition. It appears that the use of EEG decreases identification and response speeds in visual tasks, mostly concerning color visibility, as well as decreases the ability to concentrate, seen in an increase of errors during the tasks. However, this deterioration in concentration and performance does not persist when the EEG equipment is removed and switched off.

**The original study of the phenomenon explained:**

The mechanisms behind the decrease in processing speeds for visual information and concentration are not yet entirely understood. However, recent studies by Magaldanha et al. (2011) suggest that the mechanism responsible for the deterioration in identification speeds in visual tasks is the amplification of the post-synaptic action potentials by EEG.

The electroencephalograph (EEG) is an instrument that measures the electrical activity of the brain. The electrical signal used in EEG is the sum of the synchronous post-synaptic action potentials of a large number of neurons. EEG allows for measurement of brain activity with very high temporal precision, millisecond by millisecond. With this temporal precision, EEG can reveal functional alterations in the dynamics of neuroelectrical activity. However, the electrical potentials measured by EEG are very weak and very diffuse, which limits the amount of spatial information, which can be collected with this method. To address the week potentials, the electrical potential measured at the scalp is amplified.

The original study: Magaldanha et al., 2011 (Study 1) studied the neuronal activity of the visual association cortex in patients suffering from two different types of visual agnosias^^[[1]](#footnote-1)^^: *Achromatopsia* – a pathology of the visual system characterized by a complete lack of color vision or an inability to recognize colors, usually present as a result of a cerebral lesion; and *Agnostic alexia* - an inability to recognize written letters/words, usually present as a result of lesions in the occipital lobe and visual associative areas. This study additionally investigated *color blindness*, which is similar to achromatopsia in that color blind persons have difficulty in color discrimination. Color blindness, however, is not caused by cerebral lesions, or by a malformation. Rather, it is caused by an anomaly of the optical nerve (the retina), which causes difficulties in distinguishing between certain colors (red and green, for example). This anomaly can be inherited or acquired.

Experiment 1 of this study consisted of a visual discrimination task involving colors and words. The aim was to understand the differences and similarities between the three above mentioned diagnoses by examining their cerebral activity in multiple regions, including visual associative cortex, using EEG. The results of the experiment were surprising. The authors noted that patients with achromotopsia and agnostic alexia tested during EEG acquisition (slightly modified to increase the synchronous post-synaptic action potentials from the neurons in the visual cortex) performed far worse than patients with the same anomalies tested behaviorally (without EEG). Their performance was also worse than that of normal subjects tested behaviorally. This discovery was completely unexpected as the study was aimed at observing post-synaptic electrical activity in different cortical regions in these patients, not behaviorally.

Moreover, the authors observed that patients suffering from Agnostic Alexia were able to discriminate and recognize only three words out of ten, which puts them below average even compared to what is generally observed for this pathology. In order to confirm that the results from Experiment 1 were not accidental, the authors replicated the experiment (Experiment 2). The authors used the same measurement parameters (slightly modified EEG) and the same visual discrimination and recognition task. The patients were not the same as those that participated in the first experiment, but they had the same diagnoses (achromatopsia, agnostic alexia and color blindness). The results observed confirmed the findings from experiment 1. The authors concluded: “*The EEG has the potential to negatively impact participant’s capacities by acting at a post-synaptic level”.*


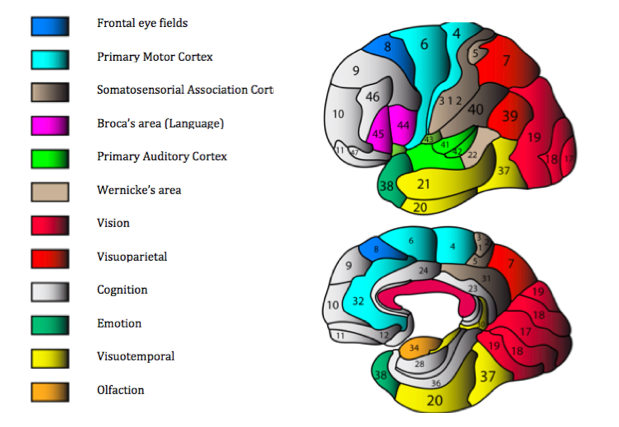


Figure 3. *Areas 5, 6, 39, and 40 are the visual associative areas implicated in the treatment of visual information.*

**Use of EEG and the present experiment:**

We believe that inducing a low-frequency electrical signal (between 3 and 30 Hz) can lead to what is known as “adaptive overburden” to the signals already present between the neurons, which leads to a partial and temporary decrease in color discrimination. By interfering with the visual associative areas, by means of electroencephalography (EEG), colors will appear less distinct. This impairment is caused by “communication cycles”. This means that the frequency of action potentials is increased only during the electrical feedback between neurons. This leads to a poorer interpretation of the signal between two neurons during neuro-feedback (Figure 4).


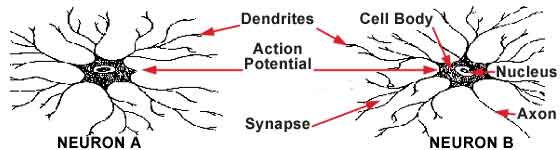


Figure 4*. An example of communication between two neurons*

**The aim of this experiment** is to replicate the results of Magaldanha et al. (2011), with a population of healthy participants (i.e. participants without achromatopsia, agnostic alexia, or color blindness). We seek to understand what causes the decrement in attention and visual abilities associated with particular EEG setups and parameters. We will use electroencephalography (EEG) during a computer-based visual discrimination task.

We will replicate the EEG setup used in the original study (Magaldanha et al. 2011) using the same parameters. With the correct parameters, you will be worse at discriminating the displayed stimuli, and your attentional capacity will be impaired. This will effectively increase the number of errors made during the task.

**ATTENTION!**

Any decline in your performance will not persist after the electroencephalograph (EEG) is switched off. Once again, we will be measuring your response times and your performance (number of errors) in a computer-based task. We think your performance will decrease when you carry out the task with the EEG, which will not be the case when you are not wearing the EEG. This will allow us to obtain (more) information on the negative impact the EEG can have. Taking part in this experiment presents no risk whatsoever.

**IMPORTANT REMINDER!!!**

**This experiment presents no risks and the interference caused in the visual associative cortex is temporary and limited.** The colors will appear less distinct only when you are wearing the EEG cap and the machine is switched on. This is due to the fact that the interference is not constant but cyclical. The electroencephalography cap (EEG) will be connected to multiple machines and your neuronal activity will be recorded.

You will not be in danger by participating in this experiment. It presents no more risk to you than a routine physical or psychological exam.

1. *Agnosia:* a cognitive dysfunction characterized by a deficit in the ability to perceive sensory stimuli, such as images or sounds. A subject with agnosia perceives stimuli, but does not process them at a logical level. *Visual agnosia*: A deficit of visual recognition. Lesions are found in the occipital lobe and visual associative areas. [↑](#footnote-ref-1)
