# Supplementary material for: Placebo-Suggestion Modulates Conflict Resolution in the Stroop Task
Source: PLoS One. 2013 Oct 9;8(10):e75701. doi: 10.1371/journal.pone.0075701 (PMC3794044; doi:10.1371/journal.pone.0075701)
Supplement: Document S3 — Positive Placebo-suggestion information read to participants in Experiment 1. Verbal information (i.e., direct suggestion) was a short version of the early document previously sent by mail (Document S1). In order to reinforce previous suggestions, verbal information was briefly repeated immediately before the experiment. (DOCX) [file pone.0075701.s003.docx]

**Supplementary material**

**(DOCUMENT S3)**

Pedro A. Magalhães De Saldanha da Gama, Hichem Slama, Emilie A. Caspar,

Wim Gevers & Axel Cleeremans

The following text is the text equally used in experiment 1. Translated (approximately) here from the original French version. The original French version can be made available upon demand to the following address:

Pedro Magalhães De Saldanha da Gama

Université Libre de Bruxelles Cp 191BR, Av. F.-D. Roosevelt, 50, 1050 Bruxelles, Belgium

Telephone number: +32 2 650 49 19

E-mail address: [pmagalha@ulb.ac.be](mailto:pmagalha@ulb.ac.be)

**DOCUMENT S3**

**EXPERIMENT 1**

(Positive Placebo-suggestion group: *verbal* information document)

NOTE: Information to be read to the participant before signing the informed consent document.

“In the present experiment, we will use an electroencephalogram (EEG) during a computer-based visual discrimination task. The EEG has the capacity to increase your performance regarding the time it takes you to discriminate the stimuli, as well as affect your ability to concentrate, reducing your error rate during the task. The mechanisms behind the increase processing speeds for visual information and increase concentration are not yet entirely understood. However, recent studies by Magaldanha et al. (2011) suggest that the mechanism responsible for the improvement in identification speeds in visual tasks is the amplification of the post-synaptic action potentials by EEG.

The aim of this experiment is to replicate the results of Magaldanha et al. (2011), with a population of healthy participants (i.e. participants without achromatopsia, agnostic alexia, or color blindness). We seek to understand how one could enhance attention and visual abilities through a particular EEG setups and parameter. To do this, we will use electroencephalography (EEG) during a computer-based visual discrimination task. The EEG setup that we will use is a replica of the original EEG setup (Magaldanha et al., 2011), with the same parameters. The EEG has the capacity to improve your performance regarding the time it takes you to discriminate the stimuli, as well as your ability to concentrate, reducing your error rate during the task.

We believe that inducing a low-frequency electrical signal (between 3 and 30 Hz) can lead to what is known as “adaptive overburden” to the signals already present among the neurons (responsible for their communication). This will lead to a partial and temporary increase in your ability to discriminate colors among stimuli. As a result of interfering with the visual associative areas of the visual cortex, by means of electroencephalography (EEG), colors will appear more distinct, as though your perception of them has been enhanced. This improvement of visual ability is caused by “communication cycles”. This means that the frequency of action potentials is increased only during electrical feedback between neurons (called "action potential"). This leads to enhanced neuro-feedback in the interpretation of the electrical signal between neuron A and neuron B (for example).

ATTENTION:

Any improvement in your performance will not persist after the electroencephalograph is switched off. Once again, we will be measuring your response times and your performance (number of errors) in a computer-based task. This will allow us to obtain (more) information on the positive impact the EEG can have on visual performance. I reconfirm that taking part in this experiment presents no risk whatsoever. We think your performance on the task will increase during the use of electroencephalography (EEG). When the same will be carrying out without the electroencephalogram (EEG), your performance will return back to normal. This experiment presents no risks whatsoever and the interference caused in the visual associative cortex is temporary and limited. Color discrimination will be increased only when you are wearing the EEG cap. This is due to the fact that the interference is not constant but cyclical. The electroencephalography cap (EEG) will be connected to multiple machines and your neuronal activity will be recorded. You will not be in danger by participating in this experiment. It presents no more risk to you than a routine physical or psychological exam or test”.
